# Untangling the reproductive puzzle: how floral traits, pollinator behavior, and breeding strategy shape reproductive success in the Himalayan medicinal herb *Phytolacca acinosa* Roxb

**DOI:** 10.3389/fpls.2025.1569420

**Published:** 2025-09-03

**Authors:** Junaid A. Magray, Bilal A. Wani, Irshad A. Nawchoo, Aijaz H. Ganie, Hanan Javid, Roof Ul Qadir

**Affiliations:** ^1^ Plant Reproductive Biology, Genetic Diversity and Phytochemistry Research Laboratory, Department of Botany, University of Kashmir, Kashmir, India; ^2^ Department of Botany, University of Kashmir, Baramulla, Jammu and Kashmir, India

**Keywords:** reproductive ecology, pollen biology, mixed mating, ambophily, evolution, conservation

## Abstract

Understanding the reproductive ecology of plants is crucial for devising strategies for their sustainable utilization and effective conservation. In this context, the present study investigates the reproductive biology of *Phytolacca acinosa*, a multipurpose medicinal herb of the Himalaya. The study aimed to examine the floral and pollen biology, breeding behavior, and pollination ecology of the species to inform conservation strategies. The results showed significant variation in floral traits across sites along an elevational gradient, reflecting reproductive adaptation to varying environmental conditions. Pollen grains were found to be tricolpate, prolate, and ellipsoidal. The pollen–ovule ratio indicates a facultative xenogamous breeding system is operative in the target species. Reproductive indices, including the outcrossing index (OCI), self-incompatibility index (SI), and selfing rate (S), along with bagging experiments, revealed that the species is both self and cross-compatible. Moreover, *Phytolacca acinosa* exhibits a mixed mating strategy, favoring geitonogamy over xenogamy. The pollination syndrome is ambophilous, with generalist pollinators—mainly from the order Hymenoptera—playing a dominant role. Based on insect visitation efficiency and pollen load on insect body, *Crabronidae* and *Apis cerana indica* were identified as the most effective pollinators. Although the inflorescence architecture, stigmatic movement, and likely apocarpous nature favor geitonogamy, the breeding system of *P. acinosa* does not conform strictly to a particular evolutionary strategy, oscillating between selfing and outcrossing. Overall, the findings offer valuable insights that will contribute to the development of effective conservation and sustainable utilization strategies for this high-value medicinal species.

## Introduction

The current geological epoch, the Anthropocene, is characterized by a rapid decline in global biodiversity caused by human activities (Ashraf et al., 2018; Chandra et al., 2021). Consequently, there is an urgent need for effective conservation strategies. Medicinal plants, especially those with high economic value, are particularly threatened in their natural habitats due to overexploitation, highlighting the need for sustainable use of these plant species (Van Wyk and Prinsloo, 2018). To protect these species, a comprehensive understanding of their reproductive biology is essential for developing conservation plans, restoring habitats, and species recovery (Verma et al., 2008; Wani et al., 2022). Identifying reproductive challenges can significantly improve conservation efforts (Sreekala, 2017). However, knowledge gaps in the reproductive biology of many medicinal plants in biodiversity-rich developing countries hinder species recovery and habitat restoration (Marbaniang et al., 2018; Tripathi et al., 2019). Therefore, expanding our scientific understanding of plant reproduction is crucial for successful conservation and restoration efforts.

Reproductive biology plays a significant role in determining the reproductive success of a plant species, thereby impacting the overall viability of populations (Carrió et al., 2009). A comprehensive understanding of various aspects of reproductive biology, including flowering phenology, breeding systems, pollination, presence and activity of pollinators, fruit set, seed germination, and seedling recruitment, is crucial for understanding species adaptations and mechanisms contributing to the decline in species population (Li et al., 2018).

The breeding system plays a crucial role in generating genetic variation and shaping the evolutionary trajectory of a species (Grant, 1971). An effective pollination system is equally essential for reproductive success, as it ensures adequate pollen transfer and seed development. Pollen traits such as viability, morphology, and exine structure, along with stigma receptivity, are key determinants of reproductive success, as they influence pollen-stigma compatibility, pollination efficiency, and fertilization timing (Shivanna and Rangaswamy, 1992; Ankush et al., 2011; Souza et al., 2016; Lone et al., 2021). This knowledge also provides insights into genetic diversity patterns, the degree of inbreeding depression, and the mechanisms driving plant-pollinator interactions (Sletvold et al., 2012; Blambert et al., 2016). Furthermore, understanding the pollination mechanisms and ecological factors that affect a species’ breeding system is vital for comprehending its life history strategy (Gamba and Muchhala, 2020). Plant-pollinator interactions are critical to the life history of flowering plants, influencing the success of sexual reproduction (Ganie et al., 2021). Since pollination is a fundamental step in plant sexual reproduction, studies on pollination ecology can offer valuable insights into the drivers and patterns of species extinction (Adler, 2007; Aguirre-Gutiérrez et al., 2015). Understanding seed biology, including germination, dormancy, dispersal ability, and seedling establishment, is also essential for improving the quality, propagation, and conservation of medicinal plants (Bareke, 2018). *Phytolacca acinosa* shows low seed germination and seedling establishment in natural habitats (Magray et al., 2023). Furthermore, while *P. acinosa* produces fleshy berries that are dispersed by frugivorous birds (Cruz et al., 2013), habitat fragmentation and a decline in disperser abundance may limit effective seed dispersal, restricting the colonization of new or suitable microsites (Cordeiro and Howe, 2003). Consequently, dispersal limitation, coupled with low seedling recruitment, contributes significantly to demographic instability and population decline over time.

Himalaya–a mega biodiversity hotspot harbors a large share of biodiversity, including a huge percentage of economically valuable medicinal flora (Tali et al., 2019; Ganie et al., 2020; Dar and Khuroo, 2020; Magray et al., 2023). However, in the past few decades, the rapidly increasing land use change, invasion of alien species, overexploitation, and climate change have posed significant threats to this rich biodiversity (Tali et al., 2015; Ganie et al., 2019; Hamid et al., 2020). Among the different factors, the unsustainable harvesting of medicinal plants is a primary driver of biodiversity loss in the Himalayas, placing numerous species on the verge of extinction (Ganie et al., 2019). One such species is *Phytolacca acinosa* Roxb., a multipurpose plant native to the Himalaya (POWO, 2024). Traditionally, it is used to treat various ailments such as sores, edema, and eye disorders (Basnet and Kalauni, 2020), and is known to possess anti-inflammatory, antibacterial, antiviral, and anticancer properties (Bailly, 2021). The plant is also used as a natural red dye for fabrics, as a vegetable, and as an ornamental species (Wu et al., 2016; Cheng et al., 2017; Liu et al., 2022). Additionally, it has been reported to have phytoremediation potential for removing heavy metals from contaminated soils (Xue et al., 2010; Zhao et al., 2014; Liu et al., 2022). All these factors have resulted in the excessive harvesting of *P. acinosa* in recent years, leading to a decline in its population size and number (Cheng et al., 2017; Magray et al., 2022, 2023). Insect herbivory, climate change, and anthropogenic activities like habitat destruction, deforestation, construction activities, trampling, and climate change further increase the pressure on populations in the wild (Ganie et al., 2019; Wani et al., 2024). Although many studies on various aspects of *P. acinosa* have been conducted (Mei et al., 2012; Krishan et al., 2022; Magray et al., 2022, 2023), studies on its reproductive biology remain lacking.

Against this backdrop, the current study investigated the reproductive biology of *P. acinosa*, particularly focusing on its floral biology, breeding strategies, and pollination ecology. Previous studies on Himalayan species such as *Actaea kashmiriana*, *Swertia thomsonii*, and *Rheum webbianum* have revealed diverse reproductive strategies, including mixed mating systems, floral plasticity, and the involvement of both insect- and wind-mediated pollination (Wani et al., 2022; Rashid et al., 2023b; Wani et al., 2023). These findings underscore the adaptive significance of reproductive flexibility in alpine and subalpine environments. Based on this context, we hypothesized that *P. acinosa* optimizes reproductive output through a complex interplay of trait adaptations, pollinator interactions, and breeding strategies, which together shape its reproductive fitness in the Himalayan environment. We further hypothesized that variations in floral traits influence pollinator attraction and behavior, while the plant’s breeding system directly impacts reproductive output. Pollinator diversity and efficiency are expected to significantly affect fruit and seed set, shaping the overall reproductive fitness of the species in its native Himalayan environment. Based on these hypotheses, the study was designed to address specific questions: (i) What are the main floral features that facilitate or hinder the reproductive success of *P. acinosa*? (ii) What type of pollination and breeding strategies are operative in *P. acinosa*, and how they influence the reproductive output of the species? (iii) What are the main pollinators of *P. acinosa*, and how are their pollination adaptations and efficiency vital in the plant-pollinator interaction? since comprehending reproductive biology is critical for developing conservation strategies. Therefore, answering these questions will aid in the successful conservation and sustainable use of *P. acinosa*. The scientific insights gained may also benefit other species in the genus *Phytolacca* and other medicinally valuable species worldwide.

## Materials and methods

### Study area

This research was conducted in the Kashmir Himalaya, India, located between latitude 33^°^20′ to 34^°^54′N and longitude 73^°^55′ to 75^°^35′E. This region is known for its rich floristic diversity, including a variety of economically important medicinal plants (Tali et al., 2019; Ganie et al., 2020). During the current study, intensive field surveys were conducted across the Kashmir Himalaya to locate the wild populations of *P. acinosa* for sampling. Four sites—Drung, Gogaldara, Gulmarg, and Doodhpathri were selected for this study based on the population size of the target species and site accessibility. The latitude, longitude, and elevation of each selected site are provided in **Table 1**. Data collection and experimentation were carried out over three consecutive years, from 2020 to 2022. The study region experiences cold winter conditions and pleasant summers, with snow cover typically from November to April. The average daily summer temperatures range between a minimum of 15°C and a maximum of 32°C, while winter temperatures range between a minimum of −4°C and a maximum of 4°C (source: India Meteorological Department, Srinagar).

**Table 1:** Latitude, longitude, and elevation (m asl) of the four study sites in the Kashmir Himalaya, India.

Study site	Latitude (N)	Longitude (E)	Elevation (m asl)
Drung	34° 2’ 16.2”	74° 24’ 39.81”	2239
Gogaldara	34° 2′ 35.41″	74° 30′ 51.3″	2448
Gulmarg	34° 2’ 16.93”	74° 23’ 45.19”	2609
Doodhpathri	33° 51’ 26.27”	74° 33’ 55.06”	2715

### Target species

**Figure 1:**
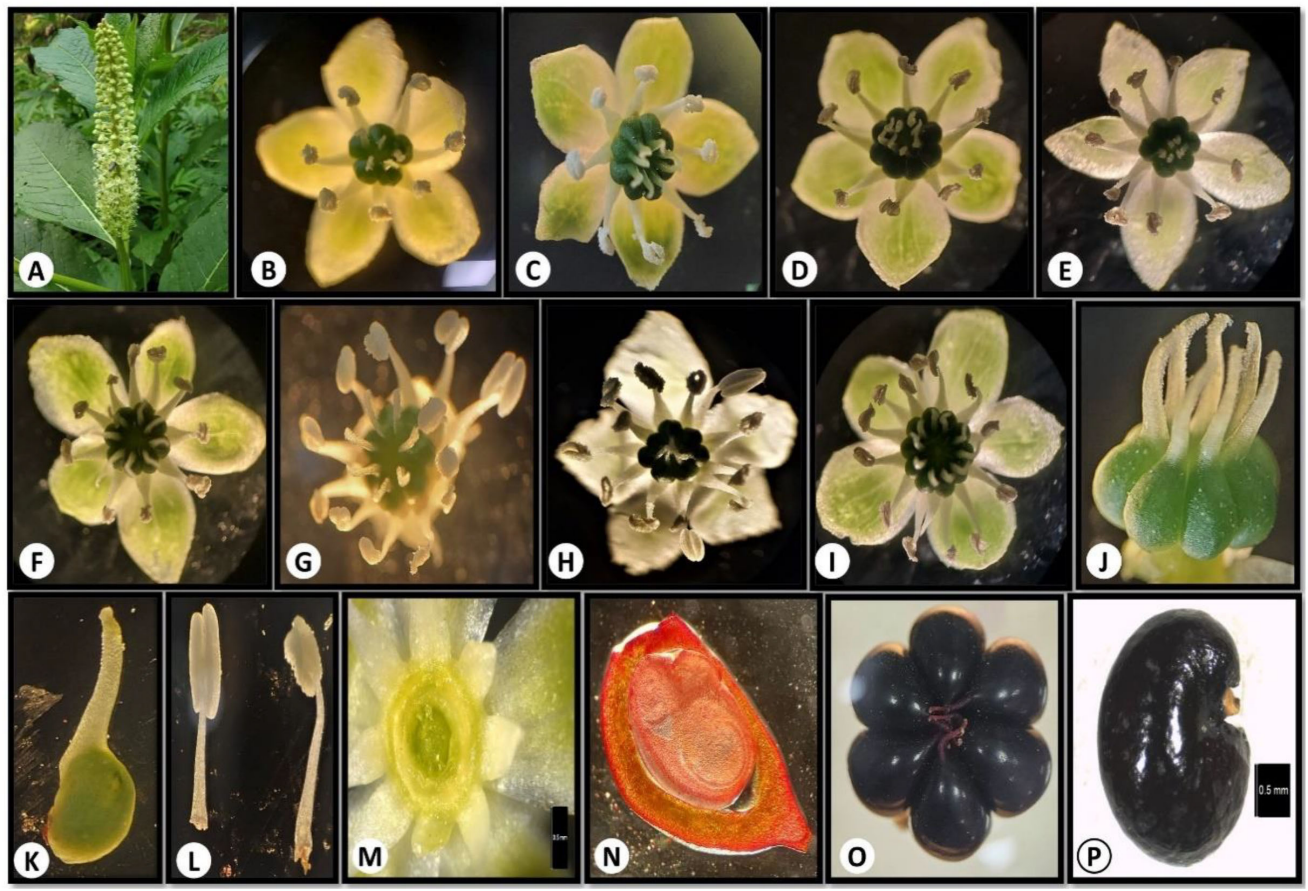
**(A-P)** Floral features of *Phytolacca acinosa*: **(A)** inflorescence; **(B-I)** variable stamen and carpel number**; (J-K)** carpel; **(L)** stamen; **(M)** nectary; **(N)** ovule; **(O)** fruit; **(P)** seed.

*Phytolacca acinosa* (Phytolaccaceae) is locally known as “Hapath wachie” and “Kafal” (Farooq et al., 2011). The species is native to the East Asian and Himalayan region (POWO, 2024) and is mostly found along roadsides, inside forests, and forest margins at an elevation of 1500–3400 m (Krishan et al., 2022; Magray et al., 2022). It is a perennial herb, stems up to 2 m tall, erect, and longitudinally grooved. The root system forms a thick, fleshy, obconic pleiocorm. Leaf petiolate, blade elliptic or lanceolate-elliptic, base cuneate, apices acuminate. Flowers are arranged in dense, erect, elongated racemes. Flowers pedicellate, bisexual; tepals 5, white or yellowish green, elliptic, ovate, or oblong, reflexed at anthesis. Nectary circular at the base of the ovary. Stamens numerous as long as tepals; filaments persistent, white, subulate, base broad; anthers elliptic. Carpels are usually 8, distinct, each terminating in a short, erect stylodium with a curved apex. Ovule campylotropous. Infructescence erect. Berry purplish black when mature. Seeds reniform, 3-angulate, smooth (**Figures 1A–P**) (FOC, 2024; Pladias, 2024).

### Floral biology

For floral trait analysis, 30 mature individuals were randomly selected and tagged at each study site. At peak flowering in July 2020–22 at Gulmarg, *P. acinosa* populations were sampled for various floral traits, including inflorescence length (IL), shape, size, and number of flowers (FN), tepal length (TPL), tepal breadth (TPB), stamen length (STL) and carpel length (CPL), pedicel length (PL), and peduncle length (PDL). Morphometric analyses were conducted on 30 fully open flowers from each site. Inflorescence length and peduncle length were measured using a measuring tape. Tepal length, tepal breadth, stamen length and carpel length, and pedicel length (PL) were measured using a digital Vernier caliper (Model Insize basic, 0–150 mm; Insize Co., India) with 0.01 mm precision (Irwin, 2000). The number of flowers per inflorescence was counted manually. The tagged plants at all selected sites were monitored regularly from the initiation to the completion of anthesis, and observations were made on floral organ movement (stigma and anthers), anthesis duration, and anther dehiscence (n = 30) were recorded (Li et al., 2018; Ganie et al., 2021). All floral parts were photographed under a Leica S9 D stereo-zoom microscope (Leica Microsystems, Wetzlar, Germany) equipped with LAS X imaging software. Destructive harvesting of plants was strictly avoided throughout the study.

### Pollen morphology and pollen viability

Pollen morphology was examined by gently tapping mature anthers to release pollen grains onto glass slides, which were then stained with 2% acetocarmine. The slides were then observed under a light microscope (Leica DM-750; Leica Microsystems, Wetzlar, Germany) (at 1000x magnification) to determine the pollen shape, size, and surface ornamentation. In addition, scanning electron microscopy (SEM) was performed to study the micromorphological features of the pollen grains. Pollen micromorphology was examined using a GeminiSEM 500 field emission scanning electron microscope (FE-SEM) (Carl Zeiss AG, Oberkochen, Germany).

Pollen viability was assessed using three standard methods: (i) Mature pollens were incubated in 1% fluorescein diacetate (FDA) solution for 2–5 minutes, with viable pollens showing fluorescent cytoplasm and non-viable ones showing non-fluorescent cytoplasm (Verma et al., 2021). (ii) Anthers ready to dehisce were squashed in aniline blue-lactophenol (1%) and examined after 15 minutes under a Leica DM-750 light microscope (Leica Microsystems, Wetzlar, Germany) (Dionne and Spicer, 1958). (iii) Mature anthers ready to dehisce were placed in 1% triphenyl tetrazolium chloride (TTC) solution for 1–2 hours, then squashed and examined under a Leica DM-750 light microscope (Leica Microsystems, Wetzlar, Germany) to determine pollen viability (Stanley and Linskens, 1974). In both cases, viable pollens appeared plump and well-stained. The pollen viability percentage for all three methods was calculated using the following formula:


$$\text{Pollen viability }(\%)=\frac{\text{Number of viable pollen grains}}{\text{Total number of pollen grains observed}}\times 100$$

### *In vitro* pollen germination

*In vitro* pollen germination was evaluated using a slightly modified version of Brewbaker and Kwack’s (1963) method. Fresh pollen grains were collected at peak anthesis from *Phytolacca acinosa* individuals growing at the Gulmarg site during 2020-2022. Pollen grains were cultured in 18 different media compositions, containing varying ratios of sucrose (10% and 20%), boric acid (500 ppm), calcium nitrate (500 ppm), magnesium sulfate (500 ppm), potassium nitrate (500 ppm), and polyethylene glycol (10%) (**Supplementary Material S1**). The cultures were incubated in cavity blocks at 21 ± 1.5°C for 12–24 hours, then slides were prepared and scanned under a microscope (Leica DM-750; Leica Microsystems, Wetzlar, Germany). A pollen tube length of greater than 1 mm was used as a criterion for pollen germination. For each treatment, five replicates were used, and a minimum of 100 pollen grains were observed per replicate.

### Stigma receptivity and pollen to ovule ratio

Stigma receptivity was assessed by fixing hand-pollinated stigmas of varying ages in Carnoy’s fixative (ethanol:glacial acetic acid, 3:1) for 3–4 hours, followed by transfer to 70% ethanol for future use. For microscopic analysis, the stigmas were stained with 1% aniline blue for 3–4 hours, following Hauser and Morrison (1964), and examined under a Leica DM750 light microscope (Leica Microsystems, Wetzlar, Germany) and a fluorescence microscope (Nikon Eclipse 80i; Nikon, Tokyo, Japan) (Wani et al., 2022). Stigmas bearing germinating pollen grains were considered receptive. Besides this, stigma receptivity was also determined by the hydrogen peroxide (H_2_O_2_) method (Dafni and Maués, 1998). In this method, stigmas were immersed in hydrogen peroxide solution for approximately 3 minutes, and the presence of oxygen bubbles was examined under a microscope (Leica DM750; Leica Microsystems, Wetzlar, Germany) as an indicator of peroxidase activity, signifying stigma receptivity. A total of 30 stigmas, collected from 10 individual plants, were tested using this method.

The pollen-to-ovule (P/O) ratio was calculated using Cruden’s (1977) method to assess the breeding system. For each site, 30 flowers (one per plant) were randomly selected. The number of pollen grains in each flower was estimated by collecting one indehiscent anther per flower, immersing it in 1 mL of detergent solution, and opening it with a lancet. Ten 10 µL subsamples were taken from the suspension and mounted on slides. Pollen grains were counted under a Leica DM-750 light microscope (Leica Microsystems, Wetzlar, Germany) at 40x. The average number of pollen grains per anther was calculated and multiplied by the total anthers per flower to estimate the total pollen production per flower. The ovary was dissected with a fine needle, and ovules were counted under a Leica S9 D stereo-zoom microscope (Leica Microsystems, Wetzlar, Germany) (Carrió et al., 2009). The P/O ratio was computed individually for each flower by dividing the total number of pollen grains by the number of ovules. The mean P/O ratio for each site was calculated from these individual values.

### Breeding system

To determine the breeding system operative in *Phytolacca acinosa*, seven bagging experiments were conducted during the peak flowering season (June–August) from 2020 to 2022 on natural plant populations growing at the Gulmarg site. A total of 150 flowers from 15 individuals (i.e, 10 flowers per plant) were randomly selected for each treatment. The experiments included: (i) apomixes **–** flowers emasculated before anthesis and bagged with butter paper; (ii) autogamy (spontaneous selfing) **–** flower buds non-manipulated and bagged with butter paper before anthesis; (iii) forced selfing **–** the stigma of flowers were manually pollinated with their own pollen grains with the help of a sterilized brush and bagged with butter paper to prevent cross-pollination; (iv) geitonogamy **–** emasculated flowers were pollinated with pollen from other flowers of the same plant and bagged with butter paper; (v) xenogamy (forced cross) **–** emasculated and bagged flowers were pollinated with pollen grains from other individuals; (vi) anemophily **–** emasculating flower buds and bagging them with mesh fabric; (vii) open pollination (control) **–** unmanipulated flowers left open for pollination (natural condition). All the flowers used were tagged, and fruit set, seed set, seed mass (per 100 seeds), and fruit dimensions (fruit length and diameter) were recorded for each bagging experiment after *ca* 50 days.

### Measurement of fruit and seed traits

The fruit dimensions were measured using a digital Vernier Caliper (Model Insize basic,0–150 mm; Insize Co., India) with 0.01 mm accuracy (Irwin, 2000). The seed mass was estimated using an electronic weighing balance (Sartorius-BSA223S, accuracy = 0.001 g). The fruit set and seed set were determined as:


$$\text{Fruit}\;\text{set}\left(\%\right)=\frac{\text{Total}\;\text{number}\;\text{of}\;\text{mature}\;\text{fruits}\;}{\text{Total}\;\text{number}\;\text{of}\;\text{flowers}\;\text{treated}}\times 100$$

$$\text{Seed}\;\text{set}\left(\%\right)=\frac{\text{Total}\;\text{number}\;\text{of}\;\text{seeds}\;\text{formed}\;}{\text{Total}\;\text{number}\;\text{of}\;\text{ovules}\;\text{borne}\;}\times 100$$

### Reproductive indices

The outcrossing index (OCI) was calculated following Dafni (1992) and Barman et al. (2018). Flower diameter, anther, and stigma positions, and the timing of stigma receptivity and anther dehiscence were recorded and compared to Dafni’s (1992) criteria to determine the breeding system in the species studied.

To determine the self-compatible/incompatible nature of the plant, the index of self-incompatibility (ISI) was calculated following Ruiz-Zapata and Arroyo (1978).


$$\text{ISI}=\frac{\text{fruit set in manual self pollination}}{\text{fruit set in manual cross pollination}}$$

ISI values ≥ 1 indicate self-compatibility; ISI = 0.2 to 1 partial self-compatibility; ISI< 0.2 mostly self-incompatible; ISI = 0 total self-incompatibility.

Selfing rate (S) was employed to assess self-pollination frequency and was calculated following Charlesworth and Charlesworth (1987).


$$\text{S}=\frac{\left(Px-Po\right)}{\left(Px-Ps\right)}$$

where *Px*= number of seeds produced in cross pollination; *Po*= number of seeds produced in open pollination; and *Ps*=number of seeds produced in self-pollination.

### Pollination mechanism

To understand the pollination ecology of *P. acinosa*, pollination censuses were conducted at peak flowering in the months of June and July during 2020–22 at Gulmarg. Various floral characteristics like the position of reproductive parts, nature of stigma, pollens and corolla were meticulously studied in 30 different flowers. To check anemophily, glycerine-smeared slides were positioned at 15, 25, and 35 cm around the target plants for 24 h, then stained with aniline blue and observed under microscope to determine the pollen number present on these slides (Wani et al., 2023). To check entomophily, we keenly observed whether insects visited the flowers, recorded the rewards (nectar and pollen) provided by the flowers, and monitored the insect behavior to assess their potential role in pollination. The flower visiting insects were captured, anesthetized in chloroform, and examined under a stereo-zoom microscope for the presence of pollens on their bodies. Representative insect specimens were identified by expert entomologists based on morphological characters. Further, pollination indices, including foraging behavior (FB), index of visitation rate (IVR), foraging speed (FS), insect visiting efficiency (IVE), and pollen load were estimated for all the pollinators to assess their contribution to pollen transfer. FB of insect visitors was determined through regular field visits. It was calculated as the time spent by a particular pollinator per inflorescence per visit using a stopwatch (Sajjad et al., 2009; Wani et al., 2022). FS was calculated as the mean number of flowers visited per unit time by a particular pollinator (Pando et al., 2011). The IVR and IVE were determined using the set methodology of Talavera et al. (2001) and Bingham and Orthner (1998), respectively. IVR provides a relative measure of pollinator visitation rate by incorporating both the frequency of visits and the activity rate of floral visitors. It was calculated using the formula:


IVR=F×AR

where, ‘F′ is the number of individuals belonging to a visiting-insect category relative to the total number of insects included in the census, and *AR* denotes the activity rate (Activity rate means the number of times an insect visits flowers included in the census).

IVE was calculated to assess the efficiency of a pollinator during a single visit. It was determined using the formula:


$$\text{IVE}=\frac{\text{Number of flowers visited by an insect in one visit}}{\text{Total number of flowers available }}$$

For estimating pollen load (pollen grains carried on the insect body), insect visitors of *P. acinosa* were captured with sweep nets, preserved in 10 ml of 70% ethanol, and vortexed to remove pollen. Pollen suspension samples (n=5) were examined under a microscope, and the total pollen load was estimated by multiplying the average pollen count per sample by the total volume (i.e, 10 ml) of the suspension (Delaplane et al., 2013). Pollen load was quantified to assess the potential of each visitor as an effective pollinator.

### Statistical analysis

Statistical analyses were conducted using IBM SPSS Statistics software, version 23.0 (IBM Corp., Armonk, NY, USA). Prior to analysis, assumptions of normality and homogeneity of variance were tested using the Shapiro–Wilk and Levene’s test, respectively. One-way ANOVA was used to assess differences in floral traits across sites, as well as to compare fruit and seed set, and other reproductive metrics across pollination treatments. *Post-hoc* Tukey’s tests were used for the comparison of means. Statistical significance was set at *p* ≤ 0.05. Data are presented as the Mean ± SE.

## Results

### Floral biology

*In Phytolacca acinosa*, anthesis exhibited notable asynchrony both within individual plants and across different individuals in the population. Flowering progressed in an acropetal sequence along the inflorescence. Anther dehiscence occurred asynchronously, beginning 2–3 days after anthesis, with pollen released through longitudinal slits in the anther wall. Before anthesis, stamens and carpels remained closely aligned; however, after anthesis, the stamens diverged away from the carpels, resulting in a pronounced spatial separation between the anthers and stigma, an apparent adaptation that promotes outcrossing. Key floral characteristics are illustrated in **Figures 1A–P**.

In the present study, analysis of variance (ANOVA) revealed significant differences (p< 0.05) in all floral traits across the study sites, except for carpel length (**Table 2**). Inflorescence length varied from 26.12 ± 1.47 cm at Doodhpathri to 33.17 ± 1.42 cm at Drung, while the number of flowers per inflorescence ranged from 151.5 ± 6.48 (Doodhpathri) to 182.4 ± 7.47 (Drung). The maximum tepal length (5.15 ± 0.12 mm) and tepal breadth (2.99 ± 0.09 mm) were recorded at Doodhpathri. Stamen length showed significant variation among sites, ranging from 3.71 ± 0.04 mm at Drung to 5.18 ± 0.07 mm at Doodhpathri, with the latter recording the maximum value.

**Table 2:** Floral traits of *Phytolacca acinosa* across the study sites.

Floral trait	SITE			
	Drung	Gogaldara	Gulmarg	Doodhpathri
TPL	3.68 ± 0.09* ^c^	5.2 ± 0.06^a^	4.23 ± 0.08^b^	5.15 ± 0.12^a^
TPB	1.79 ± 0.11^c^	3.31 ± 0.05^a^	2.26 ± 0.15^b^	2.99 ± 0.09^a^
STL (mm)	3.71 ± 0.04^c^	5.11 ± 0.06^a^	4.23 ± 0.09^b^	5.18 ± 0.07^a^
CPL (mm)	3.39 ± 0.08^a^	3.64 ± 0.07^a^	3.45 ± 0.07^a^	3.63 ± 0.05^a^
IL (cm)	33.17 ± 1.42^a^	28.04 ± 1.49^ab^	30 ± 1.55^ab^	26.12 ± 1.47^b^
FN	182.4 ± 7.47^a^	164.5 ± 7.79^ab^	171.6 ± 7.64^ab^	151.5 ± 6.48^b^
PL	0.8 ± 0.01^a^	0.73 ± 0.01^b^	0.76 ± 0.01^ab^	0.62 ± 0.01^c^
PDL	3.66 ± 0.11^b^	4.92 ± 0.12^a^	3.35 ± 0.14^b^	4.55 ± 0.13^a^

TPL, tepal length; TPB, tepal breadth; STL, stamen length; CPL, carpel length; IL, inflorescence length; FN, flower number; PL, pedicel length; PDL, peduncle length.

*Values represent mean ± Standard error (SE). Means with the different superscript letters in the same row are significantly different (*p* ≤ 0.05).

Similarly, pedicel and peduncle lengths varied significantly among sites, with the longest pedicel (0.80 ± 0.01 mm) observed at Drung and the longest peduncle (4.55 ± 0.13 mm) at Doodhpathri. Although carpel length varied slightly, ranging from 3.39 ± 0.08 mm to 3.64 ± 0.07 mm, the differences were not statistically significant (**Table 2**).

### Pollen morphology, pollen viability, and *in vitro* pollen germination

**Figure 2:**
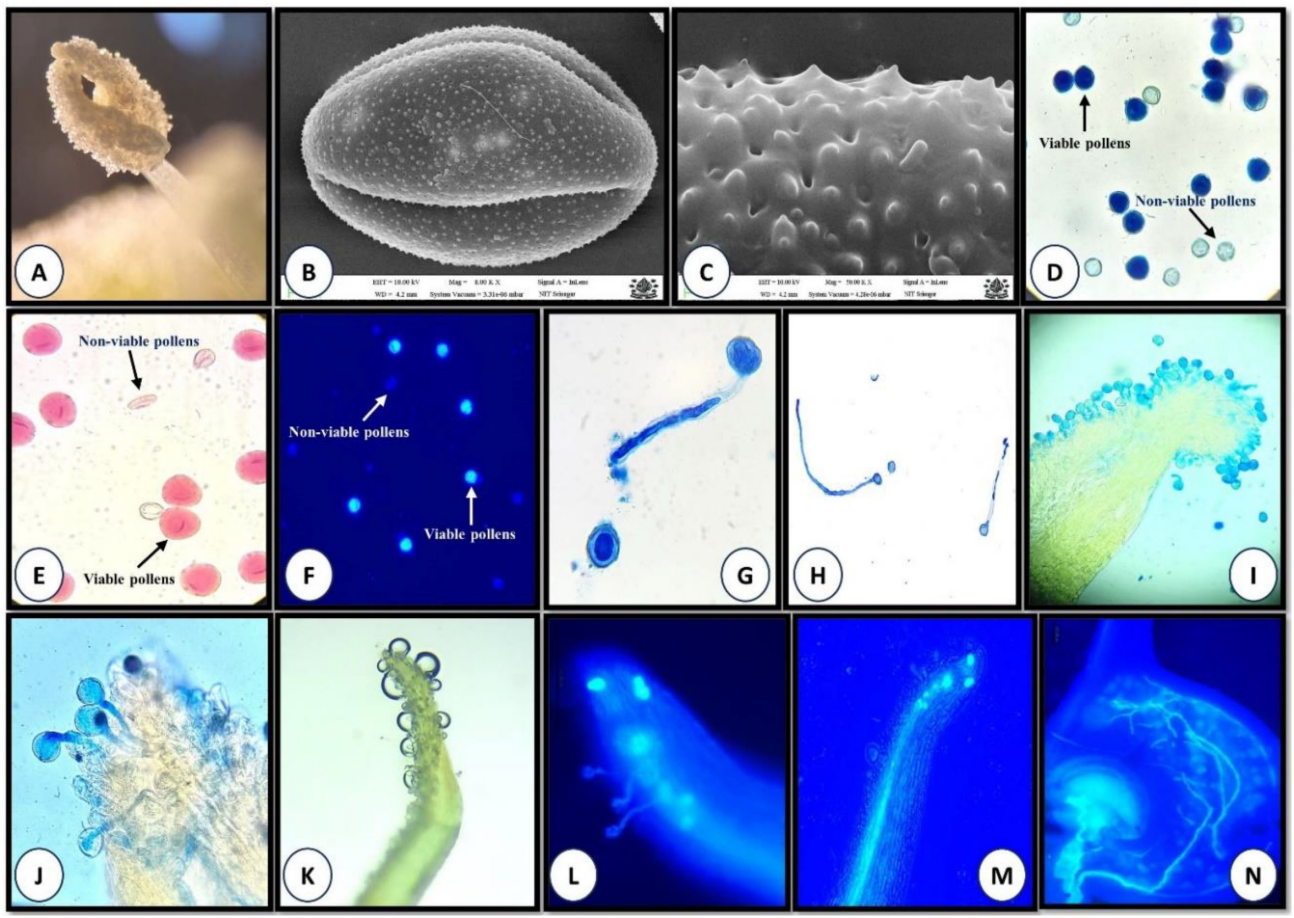
**(A-N).** Pollen biology and stigma receptivity **(A)** Anther dehiscence; **(B)** SEM graph showing tricolpate, ellipsoidal pollen; **(C)** Pollen with microechinate and perforate exine ornamentation; **(D-F)** Pollen viability (**D**-aniline blue-lactophenol, **E**-TZ, and **F**-FDA test); **(G, H)** *In vitro* pollen germination; **(I-L)** Stigma receptivity (**I** and **J**-aniline blue-lactophenol, **K**-hydrogen peroxide, and **L**-fluorescence method); **(M)** Pollen tube growing through style **(N)** Pollen tube entering ovule.

Scanning electron microscopy (SEM) and light microscopy revealed that pollen grains of *P. acinosa* are tricolpate, prolate, and ellipsoidal in shape (**Figure 2B**). The exine is thick and characterized by perforate, microechinate ornamentation (**Figure 2C**). The colpus is elongated and extends across both across both ends of the pollens (**Figure 2B**). Pollen grains are lightweight, with a polar axis measuring 26–35 µm and an equatorial axis of 21–25 µm.

Our results revealed a high percentage of pollen viability, ranging from 84.97% to 88.63%, across the four study sites. The highest viability was recorded at the Drung, with 88.63%, 87.76%, and 86.93% observed in the aniline blue-lactophenol, tetrazolium chloride, and fluorescein diacetate (FDA) tests, respectively (**Supplementary Material S2**). In contrast, the Doodhpathri site exhibited the lowest viability, with corresponding values of 85.84%, 85.19%, and 84.97% in the same three tests (**Supplementary Material S2**). Further, our findings demonstrated that sucrose, boric acid, and calcium nitrate are the key nutrients required for *in vitro* pollen germination. Among the various media compositions tested for *in vitro* pollen germination of *P. acinosa*, the highest pollen germination (64.90%) was recorded in M6 media containing sucrose (20%) and boric acid (500 ppm), followed by M4 (60.17%) and M8 (59.26%). The lowest germination (1.20%) was observed in M14, which contained sucrose (20%), magnesium sulfate (500 ppm), boric acid (500 ppm), and potassium nitrate (500 ppm) (**Supplementary Material S3**).

### Pollen-ovule ratio

The pollen and ovule numbers per flower, along with the calculated pollen-to-ovule (P/O) ratios of *Phytolacca acinosa* across the four study sites, are presented in **Supplementary Material S4**. The highest mean number of pollen grains per flower was recorded at Drung (15,733 ± 1298.1), while the lowest value was observed at Gulmarg (10,250.4 ± 1609.69). In contrast, the mean ovule number per flower showed a slight increasing trend from Gulmarg (7.03 ± 0.20) to Drung (7.80 ± 0.18). The P/O ratios ranged from 1422.43 ± 47.45 at Doodhpathri to 2017.5 ± 62.11 at Drung, indicating that *P. acinosa* exhibits a facultative xenogamous breeding system (Cruden, 1977).

### Stigma receptivity

**Figure 3:**
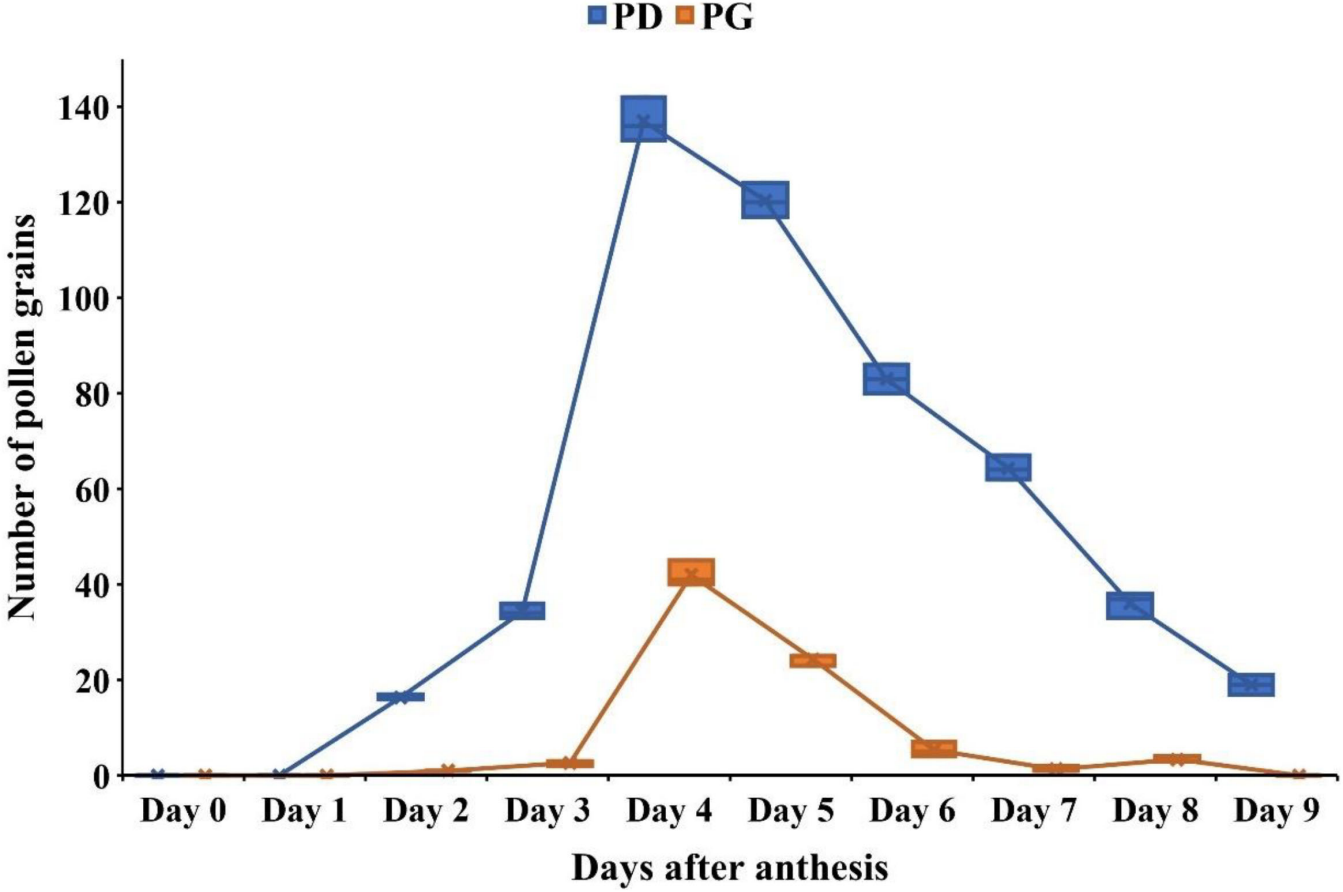
Stigma receptivity in *Phytolacca acinosa* as indicated by pollen deposition (PD) and pollen germination (PG) on the stigma surface from Day 0 (bud stage) to Day 9 after anthesis. (PD, Pollen Deposited; PG, Pollen Germinated).

Stigmas exhibited visible changes associated with receptivity, including increased moisture and curvature. On the 1^st^ day of anthesis, pollen grains were absent from the stigmatic surface. Stigma receptivity began on the 2^nd^ day after anthesis and peaked on the 4^th^ and 5^th^ day, as indicated by germinating pollen grains with tubes penetrating the stigma and growing through the style toward the ovules (**Figures 2J, L–N**, **3**). After this peak, stigma receptivity gradually declined with the increasing age of the carpels (**Figure 3**). By the ninth day after anthesis, receptivity had ended, as evidenced by the withering of the stigma and absence of germinating pollen grains (**Figure 3**).

The hydrogen peroxide test also revealed the absence of stigma receptivity at the bud stage and on the 1^st^ day of anthesis, as little to no oxygen bubbles were produced. However, on the 4^th^–5^th^ day after anthesis, at peak flowering stage, stigmatic secretions increased significantly, producing the highest number of oxygen bubbles (64.2 ± 4.2) in response to hydrogen peroxide (**Figure 2K**; **Supplementary Material S5**). Following this stage, receptivity declined rapidly, with the lowest bubble production (20 ± 2.89) observed at the withering stage (**Supplementary Material S5**).

### Breeding system and reproductive indices

The results of controlled pollination experiments revealed that no fruit was produced in emasculated and bagged floral buds, indicating the absence of apomixis in *P. acinosa*. Fruit and seed set in both self- and cross-pollination treatments suggest that the species exhibits a mixed-mating strategy and is both self and cross-compatible. Significant variation in fruit and seed set was observed among the different pollination treatments (**Table 3**). The highest fruit set (86 ± 2.94%) and seed set (82.45 ± 1.02%) was recorded in open pollination (control) followed by geitonogamy (83 ± 1.78% fruit set; 79.13 ± 0.66% seed set), xenogamy (80 ± 2.04% and 73.75 ± 0.6%), and forced selfing (75 ± 2.65% and 71.5 ± 0.65%). The lowest fruit and seed set were recorded in autonomous self-pollination (53 ± 2.86% and 60.3 ± 0.73%, respectively). In anemophily (wind pollination), a relatively lower fruit set (71 ± 2.27%) and seed set (68.58 ± 0.65%) were recorded compared to other treatments.

**Table 3:** Reproductive outcome of various bagging experiments on *Phytolacca acinosa*.

Treatment	Fruit set (%)	Seed set (%)	Seed mass per 100 seeds (g)	Fruit length (mm)	Fruit diameter (mm)
Open pollination	86 ± 2.94*^a^	82.45 ± 1.02^a^	1.29 ± 0.07^ab^	0.69 ± 0.04^a^	0.94 ± 0.04^a^
Xenogamy	80 ± 2.04^ab^	73.75 ± 0.6^c^	1.42 ± 0.07^ab^	0.65 ± 0.03^a^	0.87 ± 0.05^ab^
Autogamy	53 ± 2.86^c^	60.3 ± 0.73^e^	1.56 ± 0.08^a^	0.56 ± 0.04^a^	0.75 ± 0.03^b^
Gietnogamy	83 ± 1.78^a^	79.13 ± 0.66^b^	1.63 ± 0.09^a^	0.67 ± 0.02^a^	0.9 ± 0.04^ab^
Anemophily	71 ± 2.27^b^	68.58 ± 0.65^d^	1.14 ± 0.06^b^	0.6 ± 0.04^a^	0.8 ± 0.04^ab^
Forced selfing	75 ± 2.65^ab^	71.5 ± 0.65^cd^	1.54 ± 0.09^a^	0.64 ± 0.03^a^	0.83 ± 0.03^ab^

*Values represent mean ± Standard error (SE). Means with the different superscript letters in the same column are significantly different (*p* ≤ 0.05).

The relatively high seed set observed in geitonogamy and forced self-pollination treatments suggests that geitonogamy is the primary breeding strategy of *P. acinosa* in natural habitats. One-way ANOVA also revealed significant differences (*p ≤* 0.05) in fruit diameter, fruit length, and seed mass among pollination treatments (**Table 3**). These traits varied across treatments, with fruit length ranging from 0.56 ± 0.04 to 0.69 ± 0.04 mm, fruit diameter from 0.75 ± 0.03 to 0.94 ± 0.04 mm, and seed mass per 100 seeds from 1.14 ± 0.06 to 1.63 ± 0.09 g. The maximum fruit length (0.69 ± 0.04 mm), fruit diameter (0.94 ± 0.04 mm), and seed mass (1.63 ± 0.09 g/100 seeds) were recorded in the geitonogamy.

The self-incompatibility index (ISI), outcrossing index (OCI), and selfing rate (S) calculated for *Phytolacca acinosa* were 0.9, 4, and 1.1, respectively. An OCI value of 4 suggests that the species predominantly follows a mixed mating strategy. An ISI value of 0.9 (i.e., ≥ 0.2) indicates partial self-compatibility, while an S value of 1.1 (i.e., > 1) further corroborates the species’ self-compatible nature.

### Pollination ecology and pollination indices

*Phytolacca acinosa* exhibits an ambophilous pollination system, involving both entomophily (insect-mediated) and anemophily (wind-mediated pollination). The presence of floral traits such as a ring-shaped nectary, inflorescence structure, asynchronous anthesis, and spiny pollen exine, along with observations of insect visitation, supports its entomophilous and xenogamous nature. Anemophily was confirmed by collecting airborne pollen grains on glycerine-coated glass slides placed at different distances (15, 25, and 35 cm) from the plant. The average pollen counts recorded were 1078 ± 62, 260 ± 12, and 65 ± 5, respectively, showing a decrease in pollen number with increasing distance. Furthermore, fruit and seed set observed in insect-exclusion experiments confirmed the role of wind pollination. The fruit and seed set percentages were lower for anemophily compared to entomophily.

**Figure 4:**
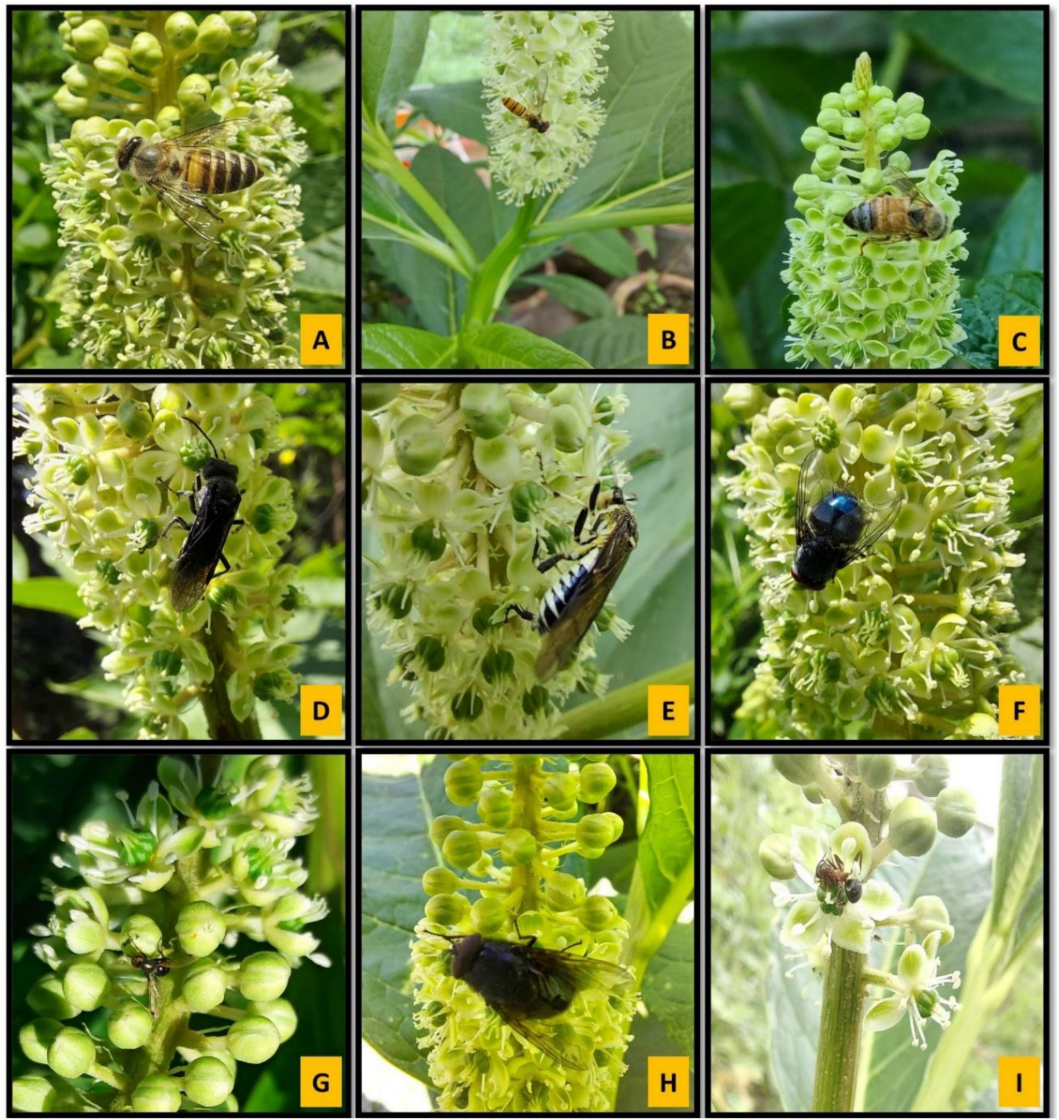
**(A-I)** Insect pollinators/visitors of *Phytolacca acinosa*: **(A)** *Apis cerana indica*; **(B)** *Episyrphus balteatus*; **(C)** *Crabronidae*; **(D)** *Symphyta* sp. 1; **(E)** *Symphyta* sp. 2; **(F)** *Calliphora vomitora*; **(G)** *Lasioglossum* sp.; **(H)** *Tachina* sp.; **(I)** *Formica* sp.

A total of 10 insect species belonging to two orders were recorded visiting the flowers (**Table 4**; **Figures 4A–I**). Hymenoptera and Diptera represented 70% and 30% of the total insect visitors, respectively. Based on pollination indices such as insect visiting efficiency and pollen load, *Crabronidae*, *Apis cerana indica*, and *Symphyta* sp.1 were identified as the most effective and dominant pollinators. In contrast, *Calliphora vomitoria* and *Calliphora* sp. were infrequent and inefficient visitors. Moreover, direct field observations on foraging behavior also showed that *Formica* sp. acted as a nectar robber, while *Tachina* sp. spent the highest time per inflorescence. The pollination indices for each insect visitor are presented in **Table 4**.

**Table 4:** Pollination indices for various insect pollinators/visitors of *Phytolacca acinosa*.

Pollinator	Foraging behavior	Insect visiting efficiency	Foraging speed	Index of visitation rate	Pollen load
*Apis cerana indica* (Hymenoptera)	71.2 ± 6.1	0.10 ± 0.005	10.1 ± 1.7	8.3 ± 1.0	4392.5 ± 117.4
*Calliphora* sp. (Hymenoptera)	50 ± 5.6	0.06 ± 0.01	4.6 ± 1.0	7.8 ± 0.9	100 ± 26
*Calliphora vomitoria* (Diptera)	73.3 ± 6.4	0.06 ± 0.008	6.3 ± 1.06	4.1 ± 0.3	54.8 ± 4.1
*Crabronidae* (Hymenoptera)	40 ± 5.1	0.16 ± 0.04	16.2 ± 2.1	12.6 ± 1.5	5031.4 ± 128
*Episyrphus balteatus* (Diptera)	36 ± 4.2	0.05 ± 0.007	5.9 ± 1.4	3.8 ± 0.6	2638.4 ± 115.3
*Formica* sp. (Hymenoptera)	81.2 ± 1	0.04 ± 0.002	4.2 ± 0.8	2.6 ± 0.2	45.2 ± 5.1
*Lasioglossum* sp. (Hymenoptera)	70 ± 6.2	0.06 ± 0.004	8 ± 1.4	3.9 ± 0.8	904 ± 20.1
*Symphyta* sp. 1 (Hymenoptera)	40 ± 4.7	0.07 ± 0.008	6.6 ± 1.3	4.4 ± 0.5	2706.2 ± 101.2
*Symphyta* sp. 2 (Hymenoptera)	38 ± 3.0	0.06 ± 0.007	6.1 ± 1.2	3.1 ± 0.4	194.3 ± 14.5
*Tachina* sp. (Diptera)	81 ± 6.4	0.04 ± 0.009	6 ± 1.3	4.9 ± 0.9	970 ± 29.44

## Discussion

This study provides comprehensive insights into the reproductive ecology of *Phytolacca acinosa*, a medicinal plant native to the Himalaya, highlighting how floral traits, pollinator interactions, and breeding mechanisms influence its reproductive success. The observed variation in floral traits, pollen viability, and pollination strategies across sites reflects the species’ ecological adaptability and reproductive plasticity in response to environmental heterogeneity.

### Floral biology

In *Phytolacca acinosa*, the morphometric analysis revealed significant variation in most floral traits across different study sites. These variations in traits can be attributed to the environmental heterogeneity across sampling sites (Tao et al., 2008; Julien and Sobrino, 2009). The floral traits, including inflorescence length and number of flowers, varied significantly (*p≤ 0.05*) along the elevation gradient, with the lower elevation site (Drung) having maximum values of these traits. At higher elevations, limited pollinator availability drives plants to produce smaller inflorescences and decrease investment in attracting pollinators, suggesting an adaptive strategy to conserve resources and promote self-fertilization (Cuartas-Hernández et al., 2019). In resource-limited environments, prioritizing resource allocation to vegetative organs becomes crucial for survival rather than investing resources in sexual reproduction (Ganie et al., 2017). The reduction in floral traits like inflorescence length, pedicel length, and peduncle length are adaptation to the strong, high-velocity winds typical of higher altitudes (Körner and Cochrane, 1985; Wani et al., 2022).

Further, floral characteristics, including tepal length, tepal breadth, and stamen length, showed a significant association with elevation. Our results are in line with Qadir et al. (2022) and Wani et al. (2023), who found that petal length and breadth varied significantly with elevation. This may be due to the heterogeneity in different ecological factors that influence the size of tepals and stamens across the different sites along the elevation gradient. Plants are sensitive to micro-environmental conditions and often adjust their reproductive traits, including tepal length, tepal breadth, and stamen length, to ensure reproductive success under varying climatic conditions.

In *P. acinosa*, the presence of sculptured exine with microechinate ornamentation suggests an adaptation for entomophily (Konzmann et al., 2019), as entomophilous species typically have pollens with a highly sculptured exine (Lumaga et al., 2006). Pollen morphology, particularly shape and exine structure, plays a crucial role in determining the pollination mechanism of a species (Ganie et al., 2016).

The study also revealed considerable variation in pollen viability across the four study sites, with the lowest viability observed at the high-elevation site Doodhpathri. This reduction in viability is likely due to rapid and extreme weather fluctuations during the reproductive stage, a common challenge for angiosperms in alpine environments (Rodríguez-Riaño and Dafni, 2007; Wani et al., 2022). The decreased pollen viability may adversely affect seed set, thereby reducing the overall fitness and fertility of the species (Khan et al., 2021).

Successful *in vitro* pollen germination requires optimal nutrients and environmental conditions. Our results showed that boric acid, sucrose, and calcium nitrate are crucial for the *in vitro* pollen germination of *P. acinosa*, with optimum temperature ranging between 20–25°C (**Supplementary Material S3**). Compounds like boric acid, sucrose, and calcium nitrate play a significant role in *in vitro* pollen germination (Parton et al., 2002; Kopp et al., 2002). In seed-bearing plants, successful fertilization and seed set depend on both pollen germination and subsequent pollen tube growth (Tiwari et al., 2017). Sucrose serves as a vital osmoregulator and nutrient, crucial for both pollen germination and tube growth, as also observed in the present study (Taylor and Hepler, 1997; Liu et al., 2013; Lin et al., 2017). Boron deficiency is known to inhibit pollen germination and cause tube bursting (Perica et al., 2001; Stino et al., 2011), while calcium is essential for the elongation and stability of pollen tubes (Hepler and Winship, 2010; Muengkaew et al., 2017). The knowledge obtained about *in vitro* pollen germination is essential for devising breeding programs and conservation strategies for the target species.

In *P. acinosa*, stigma receptivity was found to be prolonged, lasting 6–7 days, with peak receptivity occurring on the 4th–5th day of anthesis. This extended receptivity period maximizes reproductive success under varying environmental conditions (Wang et al., 2018; Wani et al., 2022). Given the broad elevational range of this species, prolonged stigma receptivity likely represents an adaptive strategy that enhances reproductive success across diverse climatic conditions.

### Breeding system

The breeding system analysis revealed that *P. acinosa* is both cross-fertile and self-compatible. The pollen-to-ovule (P/O) ratio in this species falls within the range indicative of facultative outcrossing according to Cruden (2000), which corresponds well with the outcomes of the controlled pollination experiments. This intermediate P/O ratio suggests that while *P. acinosa* primarily relies on cross-pollination; it retains the capacity for self-fertilization under limited pollinator availability (Wani et al., 2023). This reproductive flexibility may enhance its ability to adapt to varying pollination environments. Significant differences in fruit and seed set were observed across various pollination treatments (**Table 3**), with the highest fruit and seed set recorded in geitonogamy, followed by xenogamy. Geitonogamy, a form of selfing, frequently occurs in self-compatible plants that produce numerous flowers simultaneously (Harder and Barrett, 1996; Barrett, 2003). The likelihood of geitonogamous pollination increases with clonal growth, larger plant size (Barrett, 2003), and the presence of large inflorescences, where the probability of pollen transfer between flowers of the same individual is significantly higher (Ganie et al., 2016). *P. acinosa* employs a mixed mating strategy, evidenced by high fruit and seed set in both selfing and outcrossing experiments. This strategy provides reproductive assurance in fluctuating environmental conditions. However, the movement of the stigma helps prevent self-pollination, promoting cross-pollination and suggesting that the breeding system of *P. acinosa* is in an evolutionary transition between selfing and outcrossing. Similar mechanisms have been reported in *Rheum webbianum*, *Actaea kashmiriana*, and *Trillium govanianum* (Wani et al., 2022; Rashid et al., 2023a; b). Cross-pollination increases heterozygosity, enhancing genetic variability and adaptability (Maynard-Smith, 1978; Ganie et al., 2015), while self-pollination ensures seed set and survival in challenging environments (Wani et al., 2023). Consequently, many plant species have adopted a mixed mating strategy, shifting between pollination modes under limiting conditions (Goodwillie et al., 2005; Ganie et al., 2021).

### Pollination ecology

In *Phytolacca acinosa*, experimental evidence from insect exclusion trials, along with field observations, indicates the involvement of both wind and insect pollination. This dual strategy, called ambophily, likely enhances reproductive reliability, particularly under fluctuating environmental conditions and variable pollinator availability. Similar strategies have been reported in other plant species as adaptive mechanisms to maximize gene flow and reproductive efficiency (Mayer et al., 2011; Yamasaki and Sakai, 2013; Verma et al., 2021; Rashid et al., 2023b; Wani et al., 2023).

Pollinator behavior significantly influences pollen flow and genetic structure of plant populations (Shah et al., 2011). Based on pollination indices, *Crabronidae* and *Apis cerana indica* were identified as the most efficient and dominant pollinators for *P. acinosa*. Other insect visitors, such as *Episyrphus balteatus*, *Symphyta* sp., and *Tachina* sp., efficiently collect pollen while foraging, demonstrating their effectiveness as pollinators (Inouye et al., 2015). Ants, frequent floral visitors in *P. acinosa*, have also been observed as significant visitors in other species (Cursach and Rita, 2012; Rashid et al., 2023b). Most pollinators of *P. acinosa* belong to the order Hymenoptera, followed by Diptera, consistent with other studies identifying these orders as dominant pollinators (Raina et al., 2013; Wang et al., 2018). Pollinator activity peaked at noon on sunny days and was minimal or absent during late hours and cloudy days. Fluctuations in the local climate, including temperature, wind velocity, and humidity, are known to affect insect foraging behavior (Hoiss et al., 2015). The data generated for *P. acinosa* in this study will serve as a basic guide for devising appropriate restoration and conservation strategies for the proper management and sustainable utilization of this economically important medicinal plant.

### Implications for conservation and sustainable utilisation

This study provides a detailed understanding of the reproductive biology of *P. acinosa*, an important medicinal herb from the Kashmir Himalaya. The wild populations of this species are experiencing significant decline due to factors like insect herbivory, habitat degradation, deforestation, constructional activities, trampling, and climate change (Ganie et al., 2019; Magray et al., 2023; Wani et al., 2024). Human activities like road construction and deforestation have degraded much of the species’ natural habitat in the study area, leading to a reduction in population size (Ganie et al., 2019). Trampling during both vegetative and reproductive phases, caused by the movement of large herds through the region, has also contributed to a decline in the number of mature individuals. Additionally, excessive local exploitation for medicinal and other uses has further exacerbated the reduction in population size (Tali et al., 2015; Ganie et al., 2019). *Phytolacca acinosa* is also threatened by projected climate change, with currently suitable habitats predicted to become unsuitable in the near future (Wani et al., 2024). During the past five years, due to these pressures, a decline in mature individuals was observed in Gulmarg from 1,500 (2019) to 1,410 (2024), Gogaldara from 1,120 (2019) to 780 (2024), Drung from 1,246 (2019) to 975 (2024); and Doodhpathri from 960 (2019) to 780 (2024).

Our research identified various reproductive challenges in *P. acinosa*, highlighting its vulnerability. Low seed production directly restricts recruitment by reducing the number of viable seeds that can germinate, establish, and grow into reproductive adults in natural populations. The reduction in seed output can be driven by factors like low pollen viability, floral abortion, or inefficient pollination. An efficient pollination system is indispensable to deliver a suitable quantity and quality of pollen, and ultimately for effective seed maturity.

A better understanding of floral biology, including floral morphology, breeding system, pollen viability, stigma receptivity, and pollination dynamics can inform conservation interventions by identifying reproductive bottlenecks and enabling targeted actions. For example, determining whether the species is self-compatible or relies on cross-pollination can guide planting strategies or pollinator conservation efforts. Knowledge of the timing and conditions for optimal fertilization can also enhance *ex situ* seed production and germplasm conservation.

Efficient pollen transfer in this species depends on both biotic and abiotic factors. With the species relying on various pollinators for pollination, ongoing regional land-use changes and climate shifts (Hamid et al., 2020) may disrupt plant-pollinator interactions (Rather et al., 2023), further threatening the survival of the species. To ensure pollen viability for longer durations, the optimal storage conditions identified in our study should be prioritized for germplasm conservation. Additionally, the standardized pollen germination media developed could be used for *in vitro* germination of this species and potentially applied to other high-elevation plants.

Our findings contribute to effective conservation strategies, combining *in situ* and *ex situ* methods. *In situ* conservation should focus on protecting natural habitats and mitigating unsustainable anthropogenic activities, while *ex situ* efforts, such as seed collection and germination, are crucial for habitat restoration and sustainable utilization. Restoring natural populations through seedlings raised under *ex situ* conditions offers a promising approach to reviving this valuable medicinal species in the Himalaya.

## Conclusions

The present study revealed significant variation in floral traits across different habitats, reflecting the phenotypic plasticity in reproductive traits and adaptability of *P. acinosa* in varying environmental conditions. The presence of conspicuous nectary, sculptured pollens, and spatial separation between stamens and carpels are contrivances favouring entomophily and outcrossing. The fruit set observed in the insect exclusion experiment confirmed anemophily, indicating that the species is ambophilous. The reproductive indices (OCI, SI, and S) and breeding experiment results demonstrated that the species is both self and cross-fertile. Further, species exhibited a mixed matting strategy, preferring geitonogamy. The breeding system of *P. acinosa* does not follow a specific evolutionary path and is ticking between selfing and outcrossing. Therefore, the results of the study indicate that *P. acinosa* utilizes both phenotypic and reproductive plasticity to endure challenging and stressful environmental conditions in natural habitats. Finally, the information generated is highly significant and will greatly contribute to the development of effective strategies/programs for the mass cultivation, conservation, and sustainable utilization of the species.

## Data Availability

The original contributions presented in the study are included in the article/**Supplementary Material**. Further inquiries can be directed to the corresponding author.
