# Supplementary material for: Untangling the reproductive puzzle: how floral traits, pollinator behavior, and breeding strategy shape reproductive success in the Himalayan medicinal herb Phytolacca acinosa Roxb
Source: Front Plant Sci. 2025 Sep 3;16:1569420. doi: 10.3389/fpls.2025.1569420 (PMC12440930; doi:10.3389/fpls.2025.1569420)
Supplement: Supplementary file 1 [file Table1.docx]

**Supplementary Material S1:** Media compositions used for *in vitro* pollen germination

| **Medium Composition** | **Nutrients (Concentration)** | | | | | | |
| --- | --- | --- | --- | --- | --- | --- | --- |
|  | **Sucrose**  **(10%)** | **Sucrose (20%)** | **Boric acid (500ppm)** | **Magnesium sulphate (500ppm)** | **Calcium nitrate (500ppm)** | **Potassium nitrate (500ppm)** | **Polyethylene glycol**  **(10%)** |
| **M1** | **1** | **0** | **0** | **0** | **0** | **0** | **0** |
| **M2** | **0** | **1** | **0** | **0** | **0** | **0** | **0** |
| **M3** | **0** | **0** | **1** | **0** | **0** | **0** | **0** |
| **M4** | **0** | **1** | **1** | **0** | **0** | **0** | **0** |
| **M5** | **0** | **1** | **2** | **0** | **0** | **0** | **0** |
| **M6** | **0** | **2** | **1** | **0** | **0** | **0** | **0** |
| **M7** | **0** | **1** | **1** | **1** | **0** | **0** | **0** |
| **M8** | **0** | **1** | **1** | **0** | **1** | **0** | **0** |
| **M9** | **0** | **1** | **1** | **0** | **0** | **1** | **0** |
| **M10** | **0** | **1** | **1** | **1** | **1** | **1** | **0** |
| **M11** | **0** | **2** | **1** | **1** | **1** | **1** | **0** |
| **M12** | **0** | **1** | **2** | **1** | **1** | **1** | **0** |
| **M13** | **0** | **2** | **2** | **1** | **1** | **2** | **0** |
| **M14** | **0** | **1** | **1** | **1** | **0** | **1** | **0** |
| **M15** | **0** | **1** | **1** | **0** | **1** | **1** | **0** |
| **M16** | **0** | **1** | **1** | **1** | **1** | **0** | **0** |
| **M17** | **0** | **2** | **2** | **2** | **2** | **1** | **0** |
| **M18** | **0** | **0** | **0** | **0** | **0** | **0** | **1** |

1. **Absent; 1- Present (1 part); 2- Present (2 parts)**

**Supplementary Material S2:** Comparative assessment of pollen viability across 4 study sites.

| **Method** | **Population** | **Total pollens scanned** | **No. of Viable pollens** | **pollen viability (%)** |
| --- | --- | --- | --- | --- |
| **Aniline blue-lactophenal test** | Drung | 1020±30.47 ⃰ | 904±17.22 | 88.63 |
|  | Gogaldara | 1013±32.29 | 889±16.30 | 87.76 |
|  | Gulmarg | 1008±28.29 | 874±13.30 | 86.71 |
|  | Doodhpathri | 1017±14.27 | 873±10.21 | 85.84 |
| **TZ test** | Drung | 1005 ±26.80 | 882±14.82 | 87.76 |
|  | Gogaldara | 1012±27.43 | 877±13.36 | 86.66 |
|  | Gulmarg | 1014±29.37 | 871±12.39 | 85.89 |
|  | Doodhpathri | 1006±24.79 | 857±13.14 | 85.19 |
| **FDA test** | Drung | 658 ±13.80 | 572±9.82 | 86.93 |
|  | Gogaldara | 651±16.43 | 560±10.36 | 86.02 |
|  | Gulmarg | 645±14.37 | 551±11.39 | 85.43 |
|  | Doodhpathri | 652±15.79 | 574±12.14 | 84.97 |

⃰ Mean±SE

**Supplementary Material S3:** Impact of different media compositions on *in vitro* pollen germination of *Phytolacca acinosa*

**Supplementary Material S4:** Number of pollen grains and ovules per flower and the ratio Pollen-ovule (P/O) ratio of *Phytolacca acinosa* across four study sites.

| **Parameter** | **SITE** | | | |
| --- | --- | --- | --- | --- |
|  | **Drung** | **Gogaldara** | **Gulmarg** | **Doodhpathri** |
| **Mean No. of pollen grains per flower** | 15733±1298.1⃰ | 14473.5±1330.11 | 10250.4±1609.69 | 10326.9±1044.44 |
| **Mean No. of ovules per flower** | 7.80±0.18 | 7.56±0.16 | 7.03±0.20 | 7.26±0.22 |
| **Pollen-ovule ratio** | 2017.5±52.11 | 1914.48±48.13 | 1458.09±42.24 | 1422.43±37.45 |

⃰Values are Mean±SE


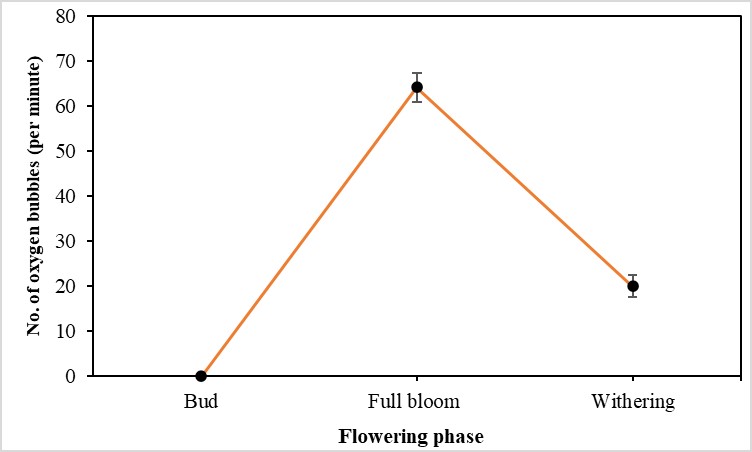


**Supplementary Material S5:** Oxygen bubble count on the stigma of *Phytolacca acinosa* at different stages.
